# Supplementary material for: Are carbon emissions trading and green financial instruments synergistic? -Comprehensive quantitative research based on content analysis
Source: PLoS One. 2024 Mar 7;19(3):e0298601. doi: 10.1371/journal.pone.0298601 (PMC10919601; doi:10.1371/journal.pone.0298601)
Supplement: S1 Data — (ZIP) [file pone.0298601.s001.zip › Data/地级市Z_F工作报告.zip!/║╙▒▒/╩»╝╥╫»╩╨/└┤╫╘╣1⁄2╓┌║┼í╛┬φ┐╦╩2╛▌═°í┐_2014.docx]

各位代表：

现在，我代表市人民政府向大会作工作报告，请予审议，并请各位政协委员和列席会议的同志提出意见。

一、2013年主要工作回顾

刚刚过去的一年，我们在省委、省政府和市委的正确领导下，紧紧围绕转型升级、跨越赶超、建设幸福石家庄的总目标，着力稳增长、调结构、抓改革、惠民生，较好地完成了市十三届人大一次会议确定的目标任务。

综合经济实力稳步提升。全市经济稳中向好，主要经济指标在全省位次前移。预计全市地区生产总值完成4863.6亿元，增长9.5%，占全省的比重由16.9%提高到17.3%。全部财政收入完成648.4亿元，增长13.1%，其中公共财政预算收入完成315.2亿元，增长15.8%，全部财政收入在9年后重返全省第一。规模以上工业增加值完成1955.4亿元，增长10.8%；实现利润640亿元，增长18%，总量居全省首位。固定资产投资完成4369.2亿元，增长20%。社会消费品零售总额完成2154亿元，增长13.7%。民营经济完成增加值3300亿元，增长11%；上缴税金407亿元，增长12%。县域经济总量占全市比重达到67.2%，新增晋州、元氏2个全部财政收入超10亿元的县（市），总数达到9个县（市）。节能减排目标超额完成，单位生产总值能耗下降4.5%。

结构调整步伐明显加快。125个省市重点项目完成投资1318亿元，超额完成年度投资计划。重点培育的装备制造、生物医药等七大主导产业投资增速达到24.2%，高于全市平均增速4.2个百分点。工业技改完成投资1271.5亿元，增长27%。战略性新兴产业增加值完成415亿元，增长22%；规上企业全部开展对标行动，对标示范企业达到86家；主营业务收入超50亿元的工业企业达到10家，其中超百亿元的企业达到5家；新增规模以上工业企业252家。服务业增加值增长10.4%，新增限上商贸企业110家，成为首批国家电子商务示范基地。省级以上研发机构达到223家，高新技术企业达到308家，实施国家和省科技项目205项，取得国内领先水平以上科技成果274项，被评为“全国科技进步先进市”。

城镇建设扎实推进。省会2011-2020年总体规划获国务院批复，中心城区扩展到287平方公里。全市在建高速公路里程157公里，新建国省干线公路142公里，太行大街、红旗大街南延具备通车条件，新客站东广场主体工程完工，轨道交通步入大规模建设阶段，建设进度在同期获批的7个城市中位居前列。新开辟公交线路19条，打通主城区断头路8条。新铺设供热主管网62公里，改造二次管网351公里，改善主城区供热面积3871万平方米，新增天然气供热面积1038万平方米。对二环路、友谊大街等主路主街进行了容貌综合整治，改造提升小街巷50条、整饰楼宇427栋，新建和提升绿地672万平方米。在北京市社科院发布的《中国城市管理报告》中，我市位列全国44个重点城市第5名。集中开展城乡建设和房地产市场整治活动，取得明显阶段性成效。正定新区迎旭大道等7条主干道竣工通车，奥体中心、职教中心等10个重大公共设施项目有序推进。正定古城启动实施保护项目38项，全国古城保护现场会在正定召开，通过了《古城保护正定宣言》。县城容貌整治集中拆违360万平方米，建设示范街道51条，新增绿地145万平方米。栾城荣获“国家卫生县城”，平山、元氏、矿区被评为“省级园林县城”，鹿泉、晋州荣获全省人居环境奖。启动岗上、铜冶等9个新市镇建设，21个重点基础设施项目建成投用。全市城镇化率达到54.4%。

“三农”工作稳定发展。粮食生产实现“十连丰”，总产达到526.8万吨，被农业部授予“全国粮食生产先进市”，藁城、赵县被评为“全国粮食生产先进县（市）”；畜牧、蔬菜、林果三大特色产业不断壮大，肉、蛋、奶产量稳定增长，市畜牧水产局被评为全国农业先进集体。实施农业产业化项目182个，完成投资47亿元，同比增长20%；市级以上重点龙头企业达到232家，农产品加工转化率达到76%。农民合作社发展到4700家，位列全省第一；新发展家庭农场和农业公司76家，都市农业园区达到48个，农村土地流转率达到18.3%，同比提高2.6个百分点。完成434个省重点村面貌改造提升任务，新建和提升农村公路506公里，硬化农村街道519公里，发展节水灌溉53万亩，解决了60万农村人口饮水安全问题。南水北调石家庄段总干渠主体工程提前竣工，配套水厂及配水管网建设顺利推进。

生态环境治理取得初步成效。大气污染防治攻坚行动全面展开，关停热电一厂和西柏坡钢铁等3家钢铁企业的6座高炉，拆除市区分散燃煤锅炉274台，对陶瓷、钙镁、水泥、化肥、焦化等行业实行限产停产，全市削减燃煤310万吨；城郊3.4万农户改烧型煤，取缔关停洗煤厂、储煤场1244家；水泥企业脱硝工程全部完成，7台火电机组完成脱硝，市区及周边14台燃煤火电机组、17台燃煤锅炉完成烟尘治理。对592个在建工地实施扬尘治理，主城区渣土车实行密闭运输；集中爆破拆除西北区域18家水泥企业，削减水泥产能940万吨；购置天然气公交车450辆，淘汰黄标车9.8万辆；油气回收任务提前一年完成；气象分析和预报预警工作进一步加强，构建起覆盖全市域的空气自动监测网络体系，主城区细颗粒物（PM2.5）平均浓度较上半年下降8.7%，成功列入“国家节能减排财政政策示范城市”。洨河综合整治取得决定性成果，水质稳定达标。大力开展植树绿化和环省会经济林建设，共造林60万亩，全市森林覆盖率达到34%。

改革开放不断深化。“四化同步”综合配套改革试验总体方案获省政府批准并启动实施。东方热电、化工化纤公司等15家国有企业改制破产基本完成。食品药品监管体制改革、卫生与计生部门整合顺利完成。积极推进事业单位分类改革。20所县级医院综合改革顺利实施，基层政府办医疗卫生机构全部实行基本药物零差率销售，“国家中医药发展综合试点市”建设成效显著。“营改增”试点正式启动，60%的乡镇纳入集中支付改革试点。扎实推进公共资源交易中心建设。小额贷款公司发展到60家，7家企业在场外市场挂牌，石家庄股权交易所成立运营。新增各类市场主体6.7万个，民营企业达到5万家。成功举办石洽会、药博会、北方旅游交易会、台湾名品博览会等一系列招商引资活动。全年实际利用外资9.8亿美元，引进市外资金836亿元，分别增长11.4%、27%。

民生保障水平持续提高。各级财政用于民生的支出达到398.6亿元，占公共预算支出的比重达到77.5%。城乡居民收入分别达到25000元和10000元，分别增长9.5%、12%。城镇新增就业10.7万人，农村劳动力转移就业6万人，城镇登记失业率3.75%。新扩建标准化公办幼儿园52所；5个县（市）区通过国家、8个县（市）区通过省义务教育基本均衡评估验收；山区教育扶贫工程56所项目学校建成投用，转移安置深山区学生3.6万名，荣获“全国教育改革创新优秀奖”。霞光大剧院建设顺利推进，丝弦剧场主体封顶；“彩色周末”、公益电影放映、送戏下乡等文化惠民活动丰富多彩；长城梦世界动漫城等一批重点文化产业项目开工建设。完成213条健身路径的安装与更新。重症肌无力诊疗中心、市八院精神卫生中心等优质卫生资源倍增工程竣工投用。新农合筹资标准由290元提高到340元，参合率达到97.6%；在全省率先实施城乡居民大病保险，城镇和农村居民最高补偿额分别达到30万元和25万元。计生家庭帮扶和流动人口计生基本公共服务均等化被确定为全国试点。连续九年提高企业退休人员基本养老金，人均每月达到1723元；提高了城乡低保、农村五保供养和孤儿养育标准，在全省率先实施贫困重度残疾人生活补贴制度。两次发放临时价格补贴，惠及低收入群体26万人。清理追回拖欠农民工工资3.9亿元。开工建设保障性住房3.8万套，竣工2.6万套，分配入住2.2万套。110综合警务服务站防控作用充分发挥，刑事发案率下降17.9%。高度重视安全生产工作，各类事故同比下降18.3%。持续开展食品药品安全专项整治行动。国防动员和双拥共建深入开展，妇女儿童、民族宗教、外事侨务、防震救灾、残疾人、档案、人防、老龄等各项事业都取得了新成绩。

政府自身建设不断加强。严格落实中央“八项规定”和党政机关厉行节约反对浪费条例。分三批削减下放行政审批事项103项，市本级精简到87项。行政服务中心绿色通道、并联审批机制不断完善，限时办结率达到100%。深入开展民主评议活动，查处损害发展环境的案件673件，给予效能责任追究592人。清理“吃空饷”人员3229名。坚持依法行政，自觉接受市人大及其常委会的法律监督、工作监督和市政协的民主监督。对重大建议和提案进行专题研究，全年办理人大代表建议395件、政协委员提案703件，按时办复率和走访率均为100%。

总结分析我市2013年工作，绝大多数经济社会发展指标好于全省平均水平，但仍有地区生产总值、规模以上工业增加值、服务业增加值、社会消费品零售总额四项经济指标没有完成预期目标，经济社会发展还存在不少困难和问题，主要是：产业结构不合理，资源利用粗放，传统产业比重较大的问题依然突出；创新能力不强，战略性新兴产业、现代服务业支撑作用还不够强，经济发展的质量和效益不高，部分企业经营困难；县域经济实力较弱，产业特色不突出；大气污染严重，生态环境改善任务十分艰巨；政府职能转变还不到位，一些部门和工作人员服务意识差，群众不满意的问题依然存在。对此，我们将采取有力措施，认真加以解决。

回顾过去一年的工作，我们在市委的正确领导下，认真贯彻落实省委、省政府的决策部署，紧紧依靠全市人民，积极应对复杂多变的经济形势，勇于承担大气污染带来的巨大压力，经受住了严峻考验，经济社会发展取得了可喜成绩。在此，我代表市人民政府，向全市人民，向人大代表、政协委员，向各民主党派、工商联、无党派人士、人民团体和社会各界人士，向驻石解放军、武警官兵和政法干警，向中直、省直机关、企事业驻石单位，向所有为石家庄发展作出贡献的投资者、创业者、劳动者，向关心支持石家庄发展的国内外朋友，致以崇高的敬意和衷心的感谢！

二、2014年主要工作任务

今年是贯彻落实党的十八届三中全会精神、全面深化改革的第一年，是加快建设大省省会、实现跨越赶超、绿色崛起的重要一年，是完成“十二五”规划目标、率先全面建成小康社会的关键一年。做好今年工作意义重大，挑战与机遇并存。从面临挑战看，国际经济仍将延续缓慢复苏态势，不确定因素依然很多；我国经济发展进入由速度规模型向质量效益型转变时期，经济增长内生动力不足，下行压力依然较大；我市正处于转型发展的攻坚时期，面临着调整结构和治理大气污染的双重压力，需要付出巨大的成本和艰苦的努力。在新一轮发展中，区域竞争日趋激烈，我们必须以更加强烈的紧迫感和使命感，敢于迎难而上，勇于担当重任，实现新的突破。从有利条件看，我国经济经过去年的稳步调整、精准应对，开始进入稳中有进、稳中向好的新阶段。习近平总书记十分关心河北和石家庄的发展，做出了一系列重要指示，为我们指明了前进方向；党的十八届三中全会拉开了全面深化改革的序幕，新型城镇化全面启动，必将极大地激发经济社会发展的内生动力和活力；京津冀协同发展上升为国家战略，环渤海地区成为我国最具增长潜力的区域，为我市发挥比较优势，加快打造京津冀第三极提供了难得的历史机遇；省委、省政府高度重视支持省会建设，市委确定的中东西三大区域协调发展战略和“一河两岸三组团”城市发展战略全面实施，一批支撑性重大项目相继建成，我市已经具备加快发展的基础和条件。我们必须进一步解放思想，强化机遇意识、进取意识、责任意识，坚持改革统领、实施创新驱动、加速绿色崛起，努力在新一轮发展中抢占先机、奋发有为、乘势而上，奋力开创全市经济社会又好又快发展新局面。

今年政府工作的总体要求是：全面贯彻落实党的十八大、十八届三中全会和中央经济工作会议、中央城镇化工作会议、中央农村工作会议，以及省委八届六次全会、市委九届五次全会精神，把改革创新贯穿于经济社会发展各个领域各个环节，坚持稳中求进、好中求快的总基调，推动新型工业化、信息化、新型城镇化和农业现代化同步发展，深入实施中东西区域协调发展战略，着力推进经济结构调整，着力提高发展质量和效益，着力加强两个环境建设，着力保障改善民生，着力维护社会稳定，坚定不移走绿色崛起之路，加快转型升级、跨越赶超、建设幸福石家庄步伐，为全面完成“十二五”奋斗目标、率先在全省全面建成小康社会奠定坚实基础。

今年全市经济社会发展的主要预期目标是：地区生产总值增长10%左右，公共财政预算收入增长13%；固定资产投资增长17%，社会消费品零售总额增长13%；城乡居民收入增长10%；居民消费价格涨幅控制在4%以内，城镇登记失业率控制在4.5%以内，人口出生率控制在14.92％。以内；单位生产总值能耗下降5.6%，化学需氧量、二氧化硫、氨氮、氮氧化物排放量分别下降3.24%、2.63%、5.32%、11.67%。

实现今年发展目标，必须全力抓好以下八个方面的工作：

（一）全面深化改革，努力增强经济社会发展活力。改革是经济发展和社会进步的永恒动力。必须坚持以改革统领经济社会发展全局，正确、准确、有序、协调推进各项改革，最大程度地激活市场主体，增强发展活力，释放改革红利。

进一步深化国有企业改革。大力发展混合所有制经济，鼓励支持国有资本、集体资本、非公有资本、域外资本等交叉持股、融合发展。以市场化和股权多元化为方向，鼓励民营企业参与国有企业改革，确保完成市动力机械厂等8家企业的改制破产任务；推动常山集团、宝德担保等7家国有独资企业实施混合所有制改革。加快完善国有资产监督管理体制，以管资本为主加强国有资产监管，促进国有资本更多地投向重要行业和关键领域。推动国有企业改制上市，提高国有资本证券化水平。进一步完善现代企业制度，合理增加国企管理人员市场化选聘比例，合理确定、严格规范国有企业管理人员薪酬待遇。加强企业家队伍建设，引领企业家不断解放思想、创新发展。推进企业财务预算等重大信息公开，强化国有企业经营投资责任追究制度，确保国有资产保值增值。

深入推进农村综合改革。在保持农村土地承包关系长久不变的前提下，开展农村土地承包经营权确权登记试点工作和建制镇、乡政府所在地集体建设用地确权发证工作。坚持和完善最严格的耕地保护制度，严守耕地红线。赋予农民对承包地占有、使用、收益、流转及承包经营权抵押、担保权能。出台我市加快农村土地承包经营权流转、促进农业规模化经营的发展意见，鼓励引导农民采取转包、出租、互换、转让等方式，流转土地承包经营权，对流转面积在200亩以上的规模化经营主体给予重点扶持，力争全市土地承包经营权流转率达到20%以上。加快构建新型农业经营体系，支持发展专业大户、家庭农场、农民合作社、农业公司等新型农业经营主体。建立市县两级农村产权交易市场，促进农村产权公开规范交易流转。加快推进户籍制度改革，实行居住证制度，出台差别化落户政策，有序放开市区户口，全面放开县级市、县城和建制镇的落户限制，有序推进农业转移人口市民化。

深化财政金融体制改革。坚持事权与财权相统一，合理划分市、县支出责任，改进财政预算管理制度，建立跨年度预算平衡机制，逐步取消竞争性领域专项。大力压缩一般性支出，整合财政资金，集中财力办大事、保民生。优化财政债务结构，实施举债核准和风险等级控制，防范和化解政府债务风险。搭建政银企合作平台，促进金融机构加大对“三农”、重点项目、小微企业和公共基础设施的信贷投放。支持符合条件的民间资本依法设立民营银行、金融租赁公司和消费金融公司。加快农村信用社股份制改造，组建一批农村商业银行。支持设立小额贷款公司和村镇银行。探索建立以园区为单位的信用保障机制，增强园区企业融资能力。鼓励支持中小企业到新三板市场融资。每个县（市）、区选择3家以上重点企业进行扶持培育，力争早日挂牌上市，年内争取全市新增上市企业10家以上。

深化激发市场主体活力改革。把发挥市场配置资源的决定性作用和更好地发挥政府的作用有机结合起来，市场能发挥作用的事项，一律放给市场；市场能有效调节的经济活动，一律取消审批。坚持权利平等、机会平等、规则平等，全面落实支持民营经济发展的“新36条”，在税费、融资、服务等方面，废除对民营经济的一切不合理规定。建立负面清单制度，各类市场主体依法平等进入清单之外领域。支持非国有资本大力发展高新技术产业和新兴服务业，进入基础设施、公共事业等领域。深化商事登记制度改革，改“先证后照”为“先照后证”，实行投资主体零限制、注册登记零收费和注册资本认缴登记制，放宽注册资本限制和住所登记条件，将企业年检制度改为年报公示制度。鼓励全民创业，筹建民营企业服务中心，制定扶持小微企业发展的专项政策，激发全民创业热情。推进政务诚信、商务诚信、社会诚信和司法公信建设，加快建立覆盖全市企业和个人的信用信息体系，依法维护市场经济秩序。认真组织好第三次全国经济普查工作。

全面构建开放型经济新体制。适应经济全球化新形势，推动对内对外开放相互促进、引进来和走出去更好结合。拓宽开放领域，推进金融、教育、文化、医疗等服务业有序开放，放开育幼养老、建筑设计、会计审计、商贸物流、电子商务等服务业领域外资准入限制。加快空港综合保税区建设进度，加大基础设施、海关和入区项目建设力度，争取近期获得国家批复。实施“走出去”战略，优化对外投资审批流程，搞好对外投资信息服务，引导钢铁、建材等过剩产能有序向境外转移，年内新增境外投资企业15家以上。强化外贸企业分类扶持，对进出口额超5000万美元的大型企业实行“一对一”帮扶，对中小微企业加强政策和实务培训，鼓励生产、商贸、物流企业联手拓展国际营销网络和建立品牌。增加能源原材料、先进技术设备和关键零部件进口，全年外贸进出口总值增长5%以上。

（二）深入实施工业强市战略，加快推进工业转型升级。工业是立市之基、强市之本。必须坚定不移地走新型工业化道路，进一步加大对产业升级、技术升级和产品升级的支持力度，加快构建现代工业体系，全面提高工业经济运行质量和效益。

大力发展战略性新兴产业。坚持“无中生有”，以大项目为牵引、大企业为龙头、科技创新为支撑，整合设立战略性新兴产业发展资金，重点支持生物制药、电子信息、高端装备制造、新能源汽车等战略性新兴产业，努力扩大规模、打造亮点。加快建设高新技术开发区、经济技术开发区、正定新能源汽车产业园等六大战略性新兴产业园区，鼓励到园区投资重大新兴产业项目。集中培育在全省乃至全国有重大影响的企业，重点支持石药集团、欣意电缆、中电科导航等11家工业企业尽快成为全市重大支撑企业，引领产业整体升级，举旗当帅。对进入省“百家企业”范围的企业给予重点扶持，促其尽快做大做强。加快推进格力电器、旭新光电、中航通用飞机等43个大项目建设。全市战略性新兴产业增加值增长22%以上，占规模以上工业增加值的比重达到20%以上。

推进传统工业转型升级。强化“有中生新”，综合运用市场、金融、财税、环保等调控手段，围绕钢铁行业提高品质、纺织服装行业提档升级、建材行业清洁生产、化工行业改进工艺，大力改造提升传统工业，全面提升企业竞争力。深化拓展对标行动，在企业研发、生产、销售等各环节全方位对标先进，全年争创省级以上标杆指标200项。加大技术改造力度，引导金融资本和社会资本积极参与企业技改，确保完成技改投资1200亿元以上，重点实施200项千万元以上技改项目。认真落实“个转企、小升规”扶持政策，年内新培育规上工业企业160家以上。严格执行能耗限额，坚决淘汰和化解钢铁、建材等行业的落后产能和过剩产能，全年净削减钢铁产能112万吨，力促鹿泉、平山、井陉等县（市）的水泥、钙镁企业实现转型发展。

大力提升科技创新能力。主动顺应新一轮国际科技革命潮流，进一步强化企业技术创新主体地位，用好省会科技教育资源，支持企业与驻石高校和科研院所联合组建技术开发平台，形成产业技术创新战略联盟，推进各类创新要素向企业聚集，确保新增高新技术企业30家以上，市级以上企业研发机构25家。设立科技型中小企业创新基金2000万元，支持中小企业尤其是科技型中小企业加快发展，选派一批科技特派员到企业开展科技服务。制定完善促进重大科研成果转化奖励政策，重点推进丁苯酞微囊、高性能光纤连接器等66项关键技术成果转化。加强关键技术研发，集中力量攻克一批具有自主知识产权的共性关键技术。依托市科技中心二期项目，下真功夫建设好科技大市场，确保6500平方米交易大厅年内建成投用，网上技术交易平台正式运行，形成“交易、共享、服务、交流”四位一体的强大功能，打造国内一流的产学研合作促进平台，建成科技资源统筹转化中心和科技创新综合服务基地，促进科技信息共享化、科技服务集成化、科技交易市场化、科技资源商品化和科技成果产业化。谋划建设科技成果孵化园区、国际科技合作基地、国际技术创业园，建设2个省级重大创新基地。实施“巨人计划”，积极对接国家“千人计划”和省“百人计划”，加快引进和培养一批科技领军人才和创新创业团队，努力形成一支结构合理、富有活力的创新人才队伍。

（三）打好项目和园区建设硬仗，构筑加快发展的重要支撑。投资拉动是我市经济发展的强大引擎。必须进一步强化抓项目就是抓发展，抓大项目就是抓大发展，抓一批大项目就是抓跨越发展的意识，大力实施“项目攻坚年”活动，努力在项目建设上实现新突破。

强力推进重大项目建设。优化政府投资结构，优先支持转型升级重大项目、重点基础设施和基础产业项目。以省支持的“三个一百”、市支持的“双11”龙头企业和149个攻坚项目为重点，强力推进总投资808亿元的河北光纤产业集群、石炼化百万吨轻烃综合利用等13个重大项目建设；确保总投资1090亿元的浙友机电生产基地、深国际现代综合物流港等51个项目如期开工，确保总投资789亿元的神威医药物流园、四药总部搬迁升级改造等48个项目竣工达效，力促石炼化800万吨炼油升级改造项目上半年正式投产。抓好总投资1138亿元的石药抗肿瘤新药产业化、北国奥特莱斯等50个项目前期手续准备工作，力争早日启动。

加强项目建设要素保障。围绕促进重点项目及时落地、按期开工、尽快竣工，全力保障土地、资金及路水电气等要素需求。用足用好省支持重大项目建设用地政策，建立用地指标安排与用地进度、供地率、补充耕地挂钩的激励机制，确保全部组卷、全部批复，对当年不能按时开工的项目收回建设用地指标。加快平山、灵寿、行唐、井陉、赞皇等山区县岗坡次地开发，加大土地整理力度，确保完成补充耕地6万亩以上。盘活闲置建设用地，开展土地利用动态巡查工作，对停产企业“腾笼换鸟”，对低效企业兼并重组。加快县级和各类园区融资平台建设，大力推广联保联贷、股权质押等金融和信贷产品，努力拓宽项目建设融资渠道。

加快重大项目谋划和招商。围绕我市产业结构调整方向和战略性新兴产业、现代服务业的发展，抓紧谋划一批符合绿色发展要求的战略性、支撑性产业项目，力争市级重点项目储备达到2000项以上。加快引进一批立市立县大项目，年内，正定、栾城、藁城、鹿泉、高新区党政主要领导至少每人引进1个10亿元以上的项目，其他县（市）、区党政主要领导至少每人引进1个5亿元以上的项目。抓住京津冀协同发展机遇，积极承接京津高新技术产业、先进制造业、物流园区、专业市场和科教机构转移。创新招商引资方式，实施委托招商、产业链招商、以商招商，面向重点区域，面向世界500强、中国500强，广泛开展上门招商，努力在寻求战略合作伙伴、重大科技成果转化上实现新突破。深化与百家央企、百家民企、百所院校的合作，精心组织好石洽会、药博会等重大经贸活动，力争使更多大项目、好项目落户我市。全年实际利用外资增长5%，引进市外资金增长15%。

提升产业园区发展水平。围绕创建“千亿级园区”，实施园区基础设施提升工程，全年投入23.7亿元，加快装备制造基地新建110千伏变电站工程、经济技术开发区世纪大道等37个园区重大基础设施项目建设，省级以上园区基本实现“七通一平”。完善园区管理体制和运行机制，提升项目审核、财税扶持、金融政策等软件服务水平，为企业发展创造良好环境。积极创建“国别型”、“区域型”、“产业型”特色园区，着力培育园区主导产业，推动产业集聚、集群发展。实施开发区产业倍增三年推进计划，力促高新技术开发区主营业务收入向3000亿元，经济技术开发区、循环化工园区、鹿泉经济开发区、装备制造基地向1000亿元，绿岛火炬开发区等5个园区向500亿元目标迈进。

（四）大力优化服务业结构，力促现代服务业超常发展。现代服务业是省会经济发展水平的重要标志。必须坚持做大总量与优化结构并重，加快推进服务业发展提速、比重提高、层次提升，力争到2015年服务业比重达到50%。

发展壮大生产性服务业。以现代物流业、金融服务业、信息服务业、交通运输业、商务服务业为重点，促进服务业与制造业融合发展。加快推进城市共同配送体系、内陆港国际保税物流园区等12个重点物流项目建设，突出抓好石家庄南部综合物流产业聚集区、正定商贸和西北（鹿泉）物流3大省级物流聚集区建设，打造全国重要物流节点城市。大力发展服务外包经济，力争世纪互联河北数据中心、IT服务外包基地扩建等项目尽快开工。以建设区域性金融中心城市为目标，争取中国进出口银行在我市设立分支机构，力促国内外金融机构总部后台服务中心入驻省会，努力打造中国北方金融后台服务产业基地。

加快发展生活性服务业。围绕丰富城乡居民生活，扩大服务供给，推进商贸服务业、健康服务业、家政服务业、法律服务业加快发展。以主城区中央商圈、国际贸易城商圈和石家庄新客站商圈为中心，做优做强核心商业圈，引领全市商贸发展，打造省会高端消费品牌。扶持北人集团等11家重点商贸企业扩规提质，推动金指数国际广场、新合作城市广场、塔坛国际商贸城等一批高标准城市综合体加快建设。推进“三级便民服务网络”建设，提升“双进”工程质量，增加社区服务网点，形成“居家一公里生活服务圈”。积极培育医疗护理、健身养生、康复保健等健康服务业。推进“万村千乡”工程，扩大农村连锁经营范围，形成覆盖城乡的商贸配送体系。建立中小商贸企业公共服务体系，重点支持1000家中小商贸流通企业发展。

积极发展新兴消费业态。以建设国家电子商务示范城市和国家信息消费试点城市为契机，实施全市电子商务三年推进计划。加快市商务云数据中心、天山电子商务总部经济中心、跨境贸易电子商务产业园等支撑性项目建设，引进阿里巴巴等3-5家知名电商企业，搭建优势行业电子商务服务平台。着力发展信息消费、网络交易、研发设计、文化创意、动漫游戏等新兴业态，加快4G网络建设，推进城市百兆光纤工程、县（市）宽带乡村工程和物联网应用示范工程。加快发展旅游产业，完善旅游服务体系，提升景区品位、加强形象宣传、建设精品线路，重点打造西柏坡红色旅游、正定古城和赵州桥历史文化旅游，嶂石岩、五岳寨、苍岩山等生态旅游品牌，力争全年旅游业总收入突破400亿元，增长20%以上，建设国内一流旅游目的地城市。

（五）实施新型城镇化战略，推进城镇建设上水平出品位。城镇化是现代化的必由之路。必须按照省委提出的“十要十不要”要求，研究优化城市空间布局的战略框架和重大举措，全力推进省会建设出品位、县城建设上水平、新市镇建设出形象，努力走出一条具有石家庄特色的新型城镇化之路。

全力推进主城区建设出品位。围绕增强省会辐射带动作用，深化“一河两岸三组团”发展战略，提升规划层次和水准，实现由扩张性发展向限定发展边界、完善服务功能、提高形象品位转变。围绕强化省会综合交通枢纽地位，积极推进铁路东南环线和石津、石衡、平赞高速前期工作，加快推进南绕城高速、京昆石太高速、京港澳高速改扩建工程，打造环省会120公里高速公路环线。力争开工建设石济客运专线工程。完成308国道、307国道、三环辅道、新赵公路栾城赵县段等293公里的干线公路改造提升。围绕拉开城市框架，启动南二环东延西拓和天山大街南延北展工程，加快形成南至衡井线、北至正定新区、东至藁城、西至井陉、矿区的城市快速通道；加快轨道交通建设，年内已开工项目完成总工程量的50%以上，启动3号线管线改迁，2号线一期工程完成可研批复，加快谋划1号线跨河工程；全面推进新城大道建设，确保7月1日前建成通车，加速建设全长27公里的体育大街至正定新区的快速公交系统。围绕提升城市承载能力，启动和平路高架桥西延工程，完成槐安路与西二环立交桥前期准备工作，加快推进新胜利大街、新客站配套路网工程；建成友谊大街和建华大街2条公交专用道，开辟主城区通往县（市）的公交线路5条以上。完成新客站东广场建设任务，实施西广场补建工程；启动建设西柏坡、上安电厂废热利用集中供热工程，确保新增和替代供热面积3000万平方米，到2020年，形成3.4亿平方米供热能力；建设城市生活垃圾、建筑垃圾和粪便无害化处理厂；对主城区33个路段和有条件的社区进行雨污分流改造，加快实施石津灌渠城区段雨污导排工程，提高基础设施配套能力和防灾减灾能力。围绕提高城市管理水平，加强精细化管理，强化规划执法，推动城市管理重心下移，拓展数字城管功能，建设智慧城市。继续高标准抓好城区容貌综合整治，完成民心河北线、环城水系西线和太行大街绿化提升；控制主城区开发强度，稳步推进城中村、旧城改造，探索新的改造模式，努力改善人民群众居住条件。巩固房地产市场专项整治成果，严格规范房地产市场秩序。

强力推进新区建设和古城保护。大力实施城市北跨发展战略，按照“低碳、生态、智慧”的理念，加快推进正定新区重大功能性项目和公建配套项目建设，为首批单位入驻创造条件，年内奥体中心主体竣工，综合商务中心、特教学校、新区中学投入使用，国际展览中心、石家庄经济学院、省二院新区分院等24个项目全面开工。新建续建隆兴大道等18条市政道路；启动地铁1号线新区预留工程建设，确保年底车站主体封顶；加快污水处理厂、地下水厂和地表水厂建设。秉承正确的古城保护理念，切实保护好其历史文化价值，加强古城规划管控，上半年全部完成古城保护项目规划设计。全面启动南城墙修缮、角楼修复、周汉河整治、南关村改造等十大工程，尽快恢复“千年古郡，北方雄镇”的历史风貌，打造华北平原上的文化明珠、旅游名城、经济强县。

加快县城和新市镇建设。注重城镇建设质量、生态环境和文化内涵，强化规划对县城建设的引领作用，实行经济社会发展规划、城乡总体规划和土地利用规划“三规合一”，一张蓝图干到底。实施基础设施、园林绿化、景观风貌等重点工程，提高教育、医疗、文化等公共服务水平，全面提升县城吸纳人口、集聚产业的功能。深入开展县城容貌综合整治行动，年内每个县（市）建成一条示范街道、一个特色街区、两个高标准出入口。支持平山、晋州、新乐3个区域次中心城市向中等城市迈进，赵县、无极、元氏、井陉等基础条件较好的县城向小城市发展。积极推进省会周边9个新市镇建设，全面启动道路交通等基础设施建设，力争用三年时间9个新市镇区域全部实现城镇化。

大力支持县域经济发展。按照中东西三大区域发展定位，分类指导各县（市）区明确主攻方向，找准发展突破口，大力提升县域经济整体实力。统筹财税政策、项目用地、环境容量、生产要素等资源，支持中部大力发展现代服务业、高新技术产业和现代装备制造业，东部加快实现工业突破，西部大力发展生态绿色产业。实施产业集群示范提升工程，培树品牌，增强实力，力争年收入超50亿元的产业集群达到19个，超百亿元的达到10个。支持经济技术开发区青岛啤酒、可口可乐、新乐新型节能环保机械制造等100个县域重点项目建设，确保按期开工、早日竣工。支持藁城、鹿泉等经济强县努力提升整体经济实力和综合竞争力，冲击全国百强；支持正定、栾城、井陉、晋州、元氏等财政收入超10亿元县（市），加快跨越赶超步伐，尽快跻身全省10强；支持行唐、灵寿、深泽、高邑、赞皇加快发展，壮大财源，年内财政收入跨上5亿元台阶。制定扶持资源枯竭城市发展政策，支持矿区尽快实现转型发展。加大扶贫开发攻坚力度，确保4万名扶贫对象稳定脱贫，支持平山、赞皇、行唐、灵寿尽快脱帽出列。

（六）加快发展现代农业，努力改善农村面貌。坚持把“三农”工作作为重中之重，以农业发展、农民增收、农村和谐为目标，全面落实支农惠农政策，让广大农民平等参与现代化进程、共享现代化成果。

提高农业综合生产能力。以保障粮食安全为核心，严格执行粮食生产核心保护区规划，稳定面积、依靠科技、主攻单产、提高总产，确保粮食播种面积稳定在1000万亩以上，粮食总产稳定在450万吨以上。增加粮食储备，保障粮食安全。优化生产区域布局，加强东部优质小麦、专用玉米核心区建设，创建100个万亩高产示范片；大力发展西部生态农业和特色农业，突出抓好大枣、核桃、板栗等特色产业，推广平山葫芦峪综合开发、赞皇片区开发、行唐团山红生态农业园区、灵寿慈峪万亩核桃基地建设等模式，每个山区县新建1-2个以上特色产业千亩方。支持赞皇县争创国家级生态县。发展壮大畜牧、蔬菜、林果三大优势产业，持续抓好“菜篮子工程”建设，年内新创建200个高标准集约化养殖示范场、20个市级蔬菜标准园，林果无公害生产技术推广面积达到230万亩。大力实施农业高效节水灌溉工程，新发展节水灌溉53万亩。推广农田保护性耕作、测土配方施肥等清洁生产技术，全年测土配方施肥面积达到800万亩以上。

提升农业产业化经营水平。以加快奶业振兴、打造奶业强市为目标，推进畜牧业规模化、标准化生产，重点围绕提高乳粉特别是婴幼儿配方乳粉生产水平，在行唐等养殖大县建设一批标准化奶源基地，支持君乐宝、三元等骨干乳品加工企业创建乳粉品牌。大力发展食品加工业，做大做强以双鸽、雨润等为重点的农业产业化龙头，新培育市级以上龙头企业20家以上，推进农业大市向食品大市转变。加大农业科技推广力度，建设新品种展示园8个，发展种子基地20万亩，农业机械化水平达到83%，农产品加工转化率达到77%。大力发展设施农业、都市农业、观光农业，重点抓好高新区佐美庄园、鹿泉紫藤葡萄庄园等5个农业示范园区建设。大力培育新型职业农民，鼓励发展农村合作经济，依法规范和提高合作社经营管理水平。加强质量和安全监管体系建设，切实保障农副产品质量安全。

改善农村生产生活条件。深入开展农村面貌改造提升行动，以厕所改造、饮水安全、道路硬化、垃圾处理、村庄绿化、危房改造6项工作为重点，新完成368个省重点村的改造提升。加强农田水利基本建设，完成4条中小河流治理和25座小型水库除险加固。抓好以“田路分家”为重点的农村路网建设，改造乡村公路400公里。实施农村电网改造升级工程，新建、改造10千伏线路1033公里。全面加快南水北调配套工程建设进度，确保6月底前具备通水调试条件。

（七）推进生态文明建设，努力实现环境质量持续好转。生态环境事关人民群众生命健康和省会形象。必须以超常的魄力、超常的措施、超常的责任、超常的氛围，坚决打好生态环境治理攻坚战，力争早日实现空气质量明显好转，确保PM2.5浓度下降6%，重污染天气大幅减少。

强力推进大气污染防治。以建设国家节能减排示范城市为目标，以“压煤、抑尘、控车、迁企、减排、增绿”为主线，大力压减煤炭消耗，继续禁止新上涉煤项目，加大减量替代工作力度，确保全年削减煤炭400万吨。拆除和搬迁污染企业，启动三环以内所有污染排放企业有序搬迁工作，重点推进石钢整体搬迁，完成新大东纺织印染等5家企业的搬迁改造。统筹规划、逐步推进市区内大型仓储、批发市场的外迁工作。一季度完成第二批17家水泥企业的拆除工作，削减水泥生产能力910万吨。切实抓好制药企业污染治理工作。严控机动车尾气污染，大力度淘汰剩余的5.3万辆黄标车。实施减排攻坚行动，全部拆除钢铁行业14台90平方米以上烧结机烟气旁路；全部完成大型水泥企业生产线脱硝工程；按期完成电力行业9家电厂24台燃煤发电机组脱硝和烟气旁路拆除任务；力争关停淘汰热电二厂南厂区和热电三厂小火电机组；玻璃行业完成玉晶公司4条生产线煤改气、脱硝工程。坚决抑制扬尘污染，所有在建工地实行绿色施工，所有露天矿山、采砂场实行绿色生产，关闭22家露天矿山，渣土车辆全部实现运输无尘化。综合治理农村面源污染，四组团县（市）各建设1-2个优质低硫型煤厂和覆盖所有乡村的型煤配送网络，10月底前，完成农户取暖燃煤锅炉改烧型煤、生活燃煤炉灶改用液化气灶任务，洁净煤使用率达到90%以上。加强土壤环境保护和综合治理，开展污染治理与修复试点示范。大力推行秸秆综合利用，全面禁止焚烧秸秆。完善重污染天气应急预案，切实减轻重污染天气对群众生产生活和身体健康的影响。

深化水环境综合整治。突出饮用水水源地保护，建设岗黄水库上游入境水预警监测系统和拦截导污工程，加强水库水质自动监测。建设滹沱河、沙河、磁河饮用水水源地一级保护区水质监测井，确保水质安全。有序推进重点河流在线监控系统建设，实现洨河沿线39个主要路口视频监控。按照洨河综合整治模式，年内完成汪洋沟综合整治工程。加强河流水质断面考核，严格执行水质目标责任制。加强对地下水污染防治的监管，持续开展专项检查行动，严厉打击通过渗坑渗井偷排偷放行为。坚决控制地下水超采。

实施生态修复工程。生态修复刻不容缓。以创建国家森林城市为目标，加快制定实施山水林田湖修复整体规划，着力构筑绿色生态屏障。重点实施环省会生态绿化、环省会经济林建设、太行山绿化、滹沱河等5条河流绿化工程；加快全市高速公路、国省干线绿化建设，构建市区通向各县（市）的绿色廊道；推进农田林网和村庄绿化工程，每个组团县（市）建设2-3个万亩以上的经济林示范区。全年新增造林50万亩，森林覆盖率达到35%以上。严格落实生态功能区规划，探索划定生态红线。完善生态补偿机制，实行最严格的源头保护、损害赔偿、责任追究制度，切实做到用制度保护生态环境。

切实加大环境监管力度。加强工业污染治理，实行排污总量IC卡控制制度。完善排污权交易，提高企业主动治污的积极性。31家重点排放企业全部实行驻厂监管和在线监控，7月1日起市区周边火电企业严格执行烟尘特别排放限值。建立市县乡村四级网格化监管系统，形成群防群治工作格局。落实部门监管责任和企业主体责任，依法严厉打击非法超标排放，坚决杜绝以破坏环境为代价获取企业利益的行为，让非法排污付出更大代价，让保护环境成为自觉行动。

各位代表！频发的严重雾霾天气，既是生态环境问题，更是重大民生问题，已经影响了省会形象和人民群众生活质量。我们一定以决战决胜的信心、壮士断腕的勇气、只争朝夕的精神，真刀真枪干一场，早日甩掉重污染城市“黑帽子”，全力打造天蓝地绿、山清水秀的生态环境，努力让全市人民呼吸上新鲜空气、喝上干净放心的水！

（八）加快发展各项社会事业，努力提高民生保障水平。民生幸福是我们的根本追求。必须大力优化公共资源配置，优先保障民生投入，优先安排民生项目，优先解决民生问题，让发展成果更多更公平地惠及全市人民。

推动实现更高质量就业。实施就业优先战略，重点抓好高校毕业生就业和下岗再就业工作，统筹抓好农村转移劳动力、城镇困难人员、退役军人就业。大力拓宽就业渠道，充分发挥服务业、劳动密集型和中小微企业吸纳就业的优势，努力扩大就业规模。完善扶持创业优惠政策，健全城乡均等公共就业创业服务体系。加强职业技能培训，对困难家庭、失业人员开展就业援助帮扶，确保城镇零就业家庭动态为零。年内城镇新增就业9.6万人，新增农村劳动力转移就业5.2万人。

努力提高城乡居民收入。增加城乡居民工资性收入，完善工资决定和正常增长机制，健全最低工资、工资支付保障制度和企业工资集体协商制度。改革机关事业单位津贴补贴制度。加大财政对“三农”的转移性支出，落实农业各项补贴政策；完善粮食主产区利益补偿机制，实行粮食最低价收购制度；大力发展非农产业，支持返乡农民工和种养能手创办实业。规范收入分配秩序，努力增加低收入者收入，扩大中等收入者比重。

优先发展教育事业。统筹各类教育协调发展，启动实施第二期学前教育三年行动计划，实施中小学教育质量提升工程，支持名校实行集团化办学，全面提升县域高中教育教学质量；推进中、高职教育衔接和社区教育试点工作，构建现代职业教育和终身教育体系；加强高等院校重点学科建设，提升服务地方经济发展的能力。加大职教园区、十五中整体迁建、二中三期征迁、二十四中整体改造等重点项目建设力度，力争早日投入使用。全力做好国家和省对长安、无极、正定等13个县（市）、区义务教育基本均衡评估工作，努力创建全国特殊教育改革试点市。

切实加强文化建设。以创建全国文明城市为目标，大力弘扬社会主义核心价值观，深入开展群众精神文明创建活动，提高市民文化素质。推进公共文化设施建设，谋划建设裴艳玲大戏院，确保霞光大剧院“五一”前完成主体工程，丝弦剧场下半年投入使用。进一步完善市、县、乡、村四级文化基础设施，丰富社区文化生活，继续办好“送戏下乡”、规范汉字书写艺术节、读书日等群众性文化活动。繁荣文化精品创作，力争使更多作品荣获国家级大奖。发展壮大演艺娱乐、出版印装、广电传媒、动漫游戏、文化创意等文化产业，加快长城梦世界动漫城、正定新区文化创新示范园、新乐伏羲文化园、赵县世界名桥博览中心等重点项目建设，大力支持县域特色文化产业发展，提高文化产业规模化、集约化、专业化水平。切实做好古城古镇、古村落、古民居和文物保护工作。积极发展体育事业，升级改造正定乒乓球训练基地，打造自行车环城赛、健步行等全民健身品牌。

加快发展医药卫生事业。深化医药卫生体制改革，全面推开县级公立医院改革，扩大基本药物制度实施范围。巩固基层医改成果，力争优质服务示范卫生院达到50所，示范村卫生室达到200所。推进优质卫生资源倍增工程，确保市一院中心院区病房楼、市四院谈固院区、市五院门诊医技楼完成主体工程，加快市一院赵卜口院区建设。深化“国家中医药发展综合改革试验市”建设，提升基层中医药服务能力。开展社区家庭医生契约服务，组建480支“片医”团队，发放200万张居民健康卡。加强医务人员职业道德教育，构建和谐医患关系。稳定低生育水平，健全计划生育特殊家庭医疗养老扶助保障体系，启动实施一方是独生子女的夫妇可生育两个孩子的政策。切实抓好“全国预防和控制出生缺陷试点城市”建设，新生儿疾病筛查率达到90%以上。

健全社会保障体系。坚持保基本、兜底线、促公平、可持续，进一步完善社会保障制度。积极做好社会保险扩面工作，全年城镇职工基本养老保险参保人数达到184万人，城镇基本医疗保险参保人数达到273万人。切实做好基本医疗保险市级统筹、工伤保险省级统筹工作，年内全部县（市）基本医疗保险实现市级统筹。新农合筹资标准由340元提高到390元，参合率保持在95%以上。开工建设保障性住房和棚户区改造住房2.7万套，竣工2.3万套，分配入住2万套。加强以城乡低保为重点的社会救助体系建设。积极发展养老服务业，支持社会资金兴办养老、康复和托养机构，新增养老床位4000张。积极发展慈善事业，创建50个社区康复示范站，建成投用残疾人康复培训中心。

全面提升社会治理水平。改进社会治理方式，坚持系统治理、依法治理、综合治理和源头治理相结合，全面推进“平安石家庄”建设。建立价格调节基金，稳步推进城镇居民阶梯水价、气价制度，实现明码标价和收费公示双覆盖，保持市场物价基本稳定。建立食品药品安全风险监测网络，实施食品药品安全县创建工作，着力提高农产品质量和食品安全水平。大力推进社区标准化建设，提高社区治理和服务水平。加强安全生产管理，坚持全覆盖、零容忍、严执法、重实效，健全隐患排查治理体系和安全预防控制体系，严防各类事故发生。实行重大事项社会稳定风险评估制度，有效预防和化解社会矛盾。加强社会管理综合治理，依法规范上访行为，严厉打击网络诈骗和各种刑事犯罪活动，确保社会充满活力、和谐有序。关心支持妇女儿童工作，保障妇女儿童权益。进一步做好国防动员、双拥共建、防震减灾、民族宗教、外事侨务、档案、气象、人防、社科等各项工作，促进社会全面进步。

继续办好惠民实事。今年，我们将继续集中财力，全力办好利民惠民十件实事。提升供热质量。对全市100个老旧小区的二次管网和换热站进行改造。缓解交通拥堵。完善交通诱导系统，通过控制左转、设置单行道等交通组织形式，建立区域微循环。改革停车管理体制，优化静态交通秩序，清理规范“三车”运营。对槐安路、二环路10处路口进行渠化改造，缓解槐安路、二环路交通拥堵问题；升级改造主城区100条次干道和小街小巷，打通民生路等4条断头路。整治老旧小区环境。对100个老旧小区道路、路灯、给排水等基础设施进行改造提升。缓解“入园难”问题。新扩建50所公办标准化幼儿园，规划建设面积10万平方米，新增入园幼儿8000人。改造提升公园广场。在城区主道两侧和重要节点新建10处街旁游园，对15座公园广场进行全面升级改造。丰富市民文化生活。对全市现有的51个街道办事处社区文化活动中心进行提档升级，配备必要的文化活动器材，满足群众日益增长的精神文化需求。建设全民健身中心。启动建设规划总用地面积1.8万平方米、总建筑面积3.1万平方米的全民健身中心，年内完成主体工程。保证饮水安全。新建联村集中供水工程18处，单村集中供水工程210处，解决310个村、50万人的饮水安全问题。开展助残行动。为1000名贫困听力障碍人员免费配发助听器，为1000名贫困白内障患者免费实施复明手术，为3000名贫困精神病患者免费提供药物，为200户残疾家庭实施无障碍改造。保持残疾人专用通道畅通，方便残疾人通行。山区贫困学生免费就读。为山区6县56所学校转移安置的12356名深山区贫困家庭学生发放生活、交通补助，保障家庭经济困难学生吃、行全免费。

三、切实加强政府自身建设

改革发展的繁重任务，对政府工作提出了新的更高要求。必须加快转变政府职能，努力创新管理方式，以更加饱满的热情、更加务实的举措，提振精气神，汇聚正能量，加快建设人民满意的服务型政府。

扎实开展党的群众路线教育实践活动。深入开展以“为民、务实、清廉”为主要内容的群众路线教育实践活动，针对群众反映最强烈、最迫切、最需要解决的问题，立行立改，善做善成。坚持从市政府领导班子做起，带头深入基层调查研究，带头落实直接联系和服务群众制度，带头改进会风文风，多到困难突出、矛盾尖锐、情况复杂的地方去，真诚倾听群众呼声，真心解决群众困难。多做打基础、利长远的事情，大力弘扬以“两个务必”为核心的西柏坡精神，艰苦创业，埋头苦干，真正把心思用在办实事、抓落实、见实效上，努力增进全市人民福祉。

努力建设服务型政府。进一步转变政府职能，切实把发展经济的着力点转到为各类市场主体创造统一开放、竞争有序的市场环境上来。加大简政放权力度，落实和承接好国务院、省政府取消下放的行政审批事项，继续做好市本级精简下放工作，修订并公开行政审批事项目录，选择1个县作为增强乡镇活力的试点，把县、乡管理更直接、更有效的事项，直接下放到县、乡。加强对县（市）、区审批事项的监督指导，做到简政放权与科学监管有机结合。优化政府组织结构，启动市、县两级政府机构改革，积极稳妥推进事业单位分类改革。推行政府向社会购买服务。大力提高服务质量，真正实现全程保姆式服务，对窗口单位服务质量实行即时测评制度。

努力建设法治型政府。把遵守宪法和法律作为施政的根本原则，注重运用法治思维和法治方式，推进行政权力规范运行，切实把政府工作纳入法制轨道。自觉接受市人大的法律监督、工作监督，市政协的民主监督和社会公众的舆论监督，坚持定期向市人大常委会报告工作，向政协通报情况，认真听取各民主党派、工商联、无党派人士和人民团体的意见。深化行政执法体制改革，梳理各级政府部门行政职权，规范行政裁量权，严格依照法定权限履行职责，加强食品药品、安全生产、环境保护、劳动保障等重点领域基层执法力量。建立法律顾问制度，对涉及群众利益和公共政策的重大事项，广泛听取社会各界意见，健全公众参与、专家论证和政府决定相结合的行政决策机制，切实做到依法决策、科学决策、民主决策。

努力建设廉洁型政府。认真落实《党政机关厉行节约反对浪费条例》、《党政机关国内公务接待管理规定》、《建立和健全惩治预防腐败体系2013－2017年工作规划》，严格履行党风廉政建设责任制，坚持“一岗双责”，坚决反对和克服“四风”，严格执行“八项规定”。从严控制机构编制和财政供养人员，严控“三公”经费支出，大幅降低行政成本。高标准推进公共资源交易中心建设，实现公共资源交易公开、公平、公正。深入推进政府机关标准化建设，优化工作流程，大力提升政府工作标准化、规范化水平。加强反腐倡廉建设，认真执行领导干部报告个人事项等制度，带头做廉洁自律、勤政为民的表率。加大行政问责和惩治腐败力度，坚决纠正损害群众利益的不正之风，加强监察、审计，对腐败问题“零容忍”，严肃查处各类违纪违法案件。推行政府工作部门权力清单制度，实施管理公开、服务公开和结果公开，让权力在阳光下运行。

各位代表！我们已经踏上全面深化改革、加速绿色崛起的新征程。展望美好前景，我们豪情满怀；完成目标任务，我们信心百倍。让我们在省委、省政府和市委的坚强领导下，与全市人民一道，同心同德，攻坚克难，锐意进取，真抓实干，加快转型升级、跨越超越、建设幸福石家庄步伐，为在全省率先全面建成小康社会而努力奋斗！
